# Supplementary material for: Genomic selection for seed yield enhances flax breeding efficiency
Source: Mol Breed. 2026 Jun 2;46(6):61. doi: 10.1007/s11032-026-01684-3 (PMC13230323; doi:10.1007/s11032-026-01684-3)
Supplement: Supplementary file 1 — Supplementary Material 1 (tables) [file 11032_2026_1684_MOESM1_ESM.docx]

**Supplementary Tables**

**Table S1** Major hyperparameters of deep learning models used for genomic selection

| Hyperparameters | DNNGS | MLPGS | GraphConvGS] | GraphAttnGS | GraphSAGEGS | GraphFormer | DeepResBLUP | DeepBLUP |
| --- | --- | --- | --- | --- | --- | --- | --- | --- |
| hidden_layers | 512,256,128,64 | 1,024,512,256 |  |  |  |  | 128,64 | 128,64,32 |
| hidden_channels |  |  | 128 | 128 | 128 |  |  |  |
| num_layers |  |  | 2 | 2 | 2 |  |  |  |
| norm |  | layer |  |  |  |  |  |  |
| residual |  | TRUE |  |  |  |  |  |  |
| heads |  |  | 4 | 4 | 4 | 4 |  |  |
| hidden_mlp |  |  | 128 | 128 | 128 | 128 |  |  |
| learning_rate | 0.001 | 0.0005 | 0.0005 | 0.0005 | 0.0005 | 0.001 | 0.001 | 0.0001 |
| batch_size | 32 | 16 |  |  |  |  | 32 | 16 |
| epochs | 300 | 300 | 500 | 500 | 500 | 500 | 100 | 200 |
| input_dropout |  | 0.05 |  |  |  |  |  |  |
| dropout | 0.3 | 0.5 | 0.2 | 0.2 | 0.2 | 0.1 | 0.2 | 0.3 |
| top_k |  |  | 20 |  | 20 | 30 |  |  |
| activation | gelu | gelu |  |  |  |  |  |  |
| batch_norm | true |  |  |  |  |  |  |  |
| weight_decay | 0.0001 | 0.0015 |  |  |  | 0.001 |  | 0.0001 |
| input_dropout | 0.1 |  |  |  |  |  |  |  |
| graph_method |  |  | knn | knn | knn | knn |  |  |
| knn_metric |  |  | euclidean | euclidean | euclidean | euclidean |  |  |
| patience | 20 | 20 | 20 | 20 | 20 | 30 |  |  |
| warmup_ratio |  | 0.1 |  |  |  |  |  |  |
| grad_clip |  | 1 |  |  |  |  |  |  |
| seeds |  | 3 |  |  |  |  |  |  |
| use_huber |  | true |  |  |  |  |  |  |
| huber_delta |  | 1 |  |  |  |  |  |  |
| swa |  | true |  |  |  |  |  |  |
| swa_start |  | 0.7 |  |  |  |  |  |  |
| swa_freq |  | 1 |  |  |  |  |  |  |
| aggr |  |  |  |  | mean |  |  |  |
| gnn_type |  |  |  |  |  | SAGE |  |  |
| gnn_hidden |  |  |  |  |  | 128 |  |  |
| transformer_layers |  |  |  |  |  | 2 |  |  |
| d_model |  |  |  |  |  | 128 |  |  |
| base_model |  |  |  |  |  |  | R_RRBLUP |  |
| dl_model |  |  |  |  |  |  | MLPGS |  |
| rrblup_lambda |  |  |  |  |  |  |  | 0.001 |
| activation |  |  |  |  |  |  |  | gelu |
| use_precomputed_rrblup |  |  |  |  |  |  |  | true |
| train_rrblup_layer |  |  |  |  |  |  |  | true |
| use_batch_norm |  |  |  |  |  |  |  | true |
| use_residual_connections |  |  |  |  |  |  |  | true |

**Table S2** Summary statistics of Illumina paired-end (PE) reads generated for the test populations

| Population | No. of samples | Method | No. of PE reads per sample | % mapped to reference genome | Total size per sample (Mb) | Coverage (X) |
| --- | --- | --- | --- | --- | --- | --- |
| Combined biparental population (BMEVSU260) | 260 | WGS PE100 | 28,155,405 | 94.47 | 5,631 | 11.17 |
| Selected breeding lines and check cultivars in 2024 (BS38 + YS61) | 99 | GBS PE150 | 6,063,057 | 97.70 | 1,758 | 3.49 |
| Selected cultivars and breeding lines (subset of BP295) | 45 | WGS PE150 | 33,579,835 | 99.33 | 10,083 | 20.00 |
| Selected breeding lines (subset of BP295) | 252 | GBS PE150 | 8,484,494 | 99.82 | 2,545 | 5.05 |

WGS: whole-genome sequencing. Coverage (X) was calculated as the total read length divided by the genome size estimate of CDC Bethune v3.0 of 504 Mb. One PE read is equal to two single reads

**Table S3** Predictive abilities (PAs) and their standard deviations for seed yield (YLD) under five-fold cross-validation (CV) using BP295, CORE293 and CORE378

| Model | BP295 | | | | CORE293 | | | | CORE378 | | | |
| --- | --- | --- | --- | --- | --- | --- | --- | --- | --- | --- | --- | --- |
|  | HAP | PC | SNP | Max | HAP | PC | SNP | Max | HAP | PC | SNP | Max |
| R_RRBLUP | 0.840 ± 0.038 | 0.750 ± 0.145 | 0.837 ± 0.038 | 0.840 ± 0.038 | 0.840 ± 0.029 | 0.834 ± 0.030 | 0.832 ± 0.030 | 0.840 ± 0.029 | 0.819 ± 0.042 | 0.815 ± 0.042 | 0.814 ± 0.043 | 0.819 ± 0.042 |
| R_GBLUP | 0.787 ± 0.052 | 0.463 ± 0.169 | 0.781 ± 0.054 | 0.787 ± 0.052 | 0.738 ± 0.052 | 0.528 ± 0.090 | 0.736 ± 0.052 | 0.738 ± 0.052 | 0.663 ± 0.070 | 0.624 ± 0.110 | 0.662 ± 0.070 | 0.663 ± 0.070 |
| BRR | 0.839 ± 0.039 | 0.721 ± 0.149 | 0.812 ± 0.056 | 0.839 ± 0.039 | 0.840 ± 0.029 | 0.834 ± 0.030 | 0.832 ± 0.030 | 0.840 ± 0.029 | 0.818 ± 0.042 | 0.814 ± 0.042 | 0.814 ± 0.043 | 0.818 ± 0.042 |
| ElasticNet | 0.814 ± 0.042 | 0.744 ± 0.146 | 0.811 ± 0.047 | 0.814 ± 0.042 | 0.803 ± 0.034 | 0.844 ± 0.028 | 0.806 ± 0.035 | 0.844 ± 0.028 | 0.789 ± 0.047 | 0.819 ± 0.041 | 0.797 ± 0.045 | 0.819 ± 0.041 |
| RFR | 0.799 ± 0.052 | 0.681 ± 0.246 | 0.803 ± 0.048 | 0.803 ± 0.048 | 0.812 ± 0.036 | 0.797 ± 0.039 | 0.815 ± 0.036 | 0.815 ± 0.036 | 0.791 ± 0.043 | 0.776 ± 0.044 | 0.799 ± 0.040 | 0.799 ± 0.040 |
| LightGBM | 0.793 ± 0.054 | 0.482 ± 0.161 | 0.789 ± 0.045 | 0.793 ± 0.054 | 0.810 ± 0.040 | 0.772 ± 0.052 | 0.822 ± 0.038 | 0.822 ± 0.038 | 0.779 ± 0.045 | 0.751 ± 0.049 | 0.792 ± 0.047 | 0.792 ± 0.047 |
| XGBoost | 0.779 ± 0.051 | 0.615 ± 0.243 | 0.758 ± 0.063 | 0.779 ± 0.051 | 0.781 ± 0.049 | 0.775 ± 0.053 | 0.788 ± 0.047 | 0.788 ± 0.047 | 0.753 ± 0.048 | 0.759 ± 0.043 | 0.764 ± 0.048 | 0.764 ± 0.048 |
| MLPGS | 0.790 ± 0.048 | 0.693 ± 0.181 | 0.794 ± 0.053 | 0.794 ± 0.053 | 0.825 ± 0.037 | 0.804 ± 0.043 | 0.811 ± 0.050 | 0.825 ± 0.037 | 0.803 ± 0.050 | 0.798 ± 0.044 | 0.802 ± 0.045 | 0.803 ± 0.050 |
| DNNGS | 0.808 ± 0.063 | 0.507 ± 0.347 | 0.823 ± 0.050 | 0.823 ± 0.050 | 0.815 ± 0.051 | 0.806 ± 0.041 | 0.799 ± 0.074 | 0.815 ± 0.051 | 0.810 ± 0.046 | 0.776 ± 0.083 | 0.804 ± 0.052 | 0.810 ± 0.046 |
| GraphConvGS | 0.703 ± 0.122 | 0.252 ± 0.267 | 0.638 ± 0.189 | 0.703 ± 0.122 | 0.613 ± 0.055 | 0.594 ± 0.144 | 0.594 ± 0.092 | 0.613 ± 0.055 | 0.557 ± 0.082 | 0.521 ± 0.129 | 0.535 ± 0.102 | 0.557 ± 0.082 |
| GraphAttnGS | 0.699 ± 0.081 | 0.476 ± 0.244 | 0.631 ± 0.170 | 0.699 ± 0.081 | 0.547 ± 0.153 | 0.463 ± 0.255 | 0.551 ± 0.145 | 0.551 ± 0.145 | 0.531 ± 0.105 | 0.447 ± 0.183 | 0.471 ± 0.226 | 0.531 ± 0.105 |
| GraphSAGEGS | 0.765 ± 0.065 | 0.724 ± 0.146 | 0.760 ± 0.060 | 0.765 ± 0.065 | 0.807 ± 0.039 | 0.750 ± 0.055 | 0.783 ± 0.042 | 0.807 ± 0.039 | 0.772 ± 0.047 | 0.731 ± 0.055 | 0.763 ± 0.053 | 0.772 ± 0.047 |
| GraphFormer | 0.734 ± 0.077 | 0.728 ± 0.151 | 0.730 ± 0.071 | 0.734 ± 0.077 | 0.817 ± 0.035 | 0.783 ± 0.039 | 0.796 ± 0.039 | 0.817 ± 0.035 | 0.780 ± 0.048 | 0.758 ± 0.045 | 0.770 ± 0.047 | 0.780 ± 0.048 |
| DeepResBLUP | 0.829 ± 0.042 | 0.737 ± 0.148 | 0.828 ± 0.040 | 0.829 ± 0.042 | 0.836 ± 0.032 | 0.813 ± 0.036 | 0.829 ± 0.034 | 0.836 ± 0.032 | 0.813 ± 0.043 | 0.791 ± 0.043 | 0.812 ± 0.044 | 0.813 ± 0.043 |
| DeepBLUP | 0.821 ± 0.050 | 0.726 ± 0.156 | 0.805 ± 0.054 | 0.821 ± 0.050 | 0.828 ± 0.030 | 0.826 ± 0.031 | 0.815 ± 0.038 | 0.828 ± 0.030 | 0.805 ± 0.044 | 0.803 ± 0.043 | 0.797 ± 0.047 | 0.805 ± 0.044 |
| EnsembleGS | 0.794 ± 0.055 | 0.709 ± 0.156 | 0.792 ± 0.047 | 0.794 ± 0.055 | 0.807 ± 0.039 | 0.771 ± 0.051 | 0.819 ± 0.038 | 0.819 ± 0.038 | 0.775 ± 0.042 | 0.747 ± 0.048 | 0.785 ± 0.047 | 0.785 ± 0.047 |

**Table S4** Predictive abilities (PAs) of four test populations for seed yield (YLD) under across-population prediction (APP) using CORE293 as the training population

| Model | Marker representation | Test population | | | |
| --- | --- | --- | --- | --- | --- |
|  |  | BP295 | YS38 | BS61 | BMEVSU260 |
| R_RRBLUP | PC | 0.707 | 0.647 | 0.543 | 0.772 |
|  | HAP | 0.717 | 0.585 | 0.595 | 0.794 |
|  | SNP | 0.707 | 0.624 | 0.558 | 0.762 |
| R_GBLUP | PC | 0.586 | 0.711 | 0.521 | 0.310 |
|  | HAP | 0.522 | 0.681 | 0.592 | 0.694 |
|  | SNP | 0.499 | 0.711 | 0.521 | 0.314 |
| BRR | PC | 0.707 | 0.647 | 0.542 | 0.772 |
|  | HAP | 0.717 | 0.584 | 0.595 | 0.794 |
|  | SNP | 0.707 | 0.624 | 0.558 | 0.761 |
| ElasticNet | PC | 0.710 | 0.668 | 0.559 | 0.774 |
|  | HAP | 0.690 | 0.571 | 0.616 | 0.722 |
|  | SNP | 0.699 | 0.526 | 0.578 | 0.723 |
| RFR | PC | 0.680 | 0.658 | 0.370 | 0.738 |
|  | HAP | 0.717 | 0.470 | 0.574 | 0.681 |
|  | SNP | 0.716 | 0.393 | 0.363 | 0.627 |
| LightGBM | PC | 0.731 | 0.586 | 0.299 | 0.763 |
|  | HAP | 0.673 | 0.528 | 0.598 | 0.621 |
|  | SNP | 0.674 | 0.400 | 0.434 | 0.626 |
| XGBoost | PC | 0.696 | 0.542 | 0.249 | 0.679 |
|  | HAP | 0.692 | 0.536 | 0.466 | 0.647 |
|  | SNP | 0.612 | 0.451 | 0.440 | 0.595 |
| MLPGS | PC | 0.691 | 0.352 | 0.251 | 0.781 |
|  | HAP | 0.730 | 0.267 | 0.095 | 0.824 |
|  | SNP | 0.721 | 0.305 | 0.183 | 0.806 |
| DNNGS | PC | 0.693 | 0.698 | 0.517 | 0.777 |
|  | HAP | 0.709 | 0.590 | 0.546 | 0.808 |
|  | SNP | 0.734 | 0.648 | 0.546 | 0.789 |
| GraphConvGS | PC | 0.496 | 0.656 | -0.372 | 0.820 |
|  | HAP | 0.648 | 0.538 | -0.151 | 0.835 |
|  | SNP | 0.624 | 0.589 | -0.359 | 0.808 |
| GraphAttnGS | PC | 0.508 | 0.710 | -0.423 | 0.685 |
|  | HAP | 0.572 | 0.411 | -0.108 | 0.788 |
|  | SNP | 0.568 | 0.511 | 0.518 | 0.834 |
| GraphSAGEGS | PC | 0.646 | 0.303 | -0.005 | 0.779 |
|  | HAP | 0.719 | 0.128 | 0.284 | 0.829 |
|  | SNP | 0.728 | 0.223 | 0.188 | 0.800 |
| GraphFormer | PC | 0.697 | 0.306 | 0.168 | 0.754 |
|  | HAP | 0.718 | 0.034 | 0.171 | 0.822 |
|  | SNP | 0.692 | 0.259 | 0.196 | 0.817 |
| DeepResBLUP | PC | 0.655 | 0.574 | 0.347 | 0.781 |
|  | HAP | 0.696 | 0.598 | 0.598 | 0.789 |
|  | SNP | 0.710 | 0.648 | 0.535 | 0.784 |
| DeepBLUP | PC | 0.673 | 0.650 | 0.585 | 0.752 |
|  | HAP | 0.731 | 0.552 | 0.484 | 0.798 |
|  | SNP | 0.663 | 0.684 | 0.567 | 0.781 |
| EnsembleGS | PC | 0.728 | 0.546 | 0.236 | 0.776 |
|  | HAP | 0.702 | 0.450 | 0.553 | 0.742 |
|  | SNP | 0.653 | 0.307 | 0.380 | 0.690 |
|  | PC mean | 0.663 | 0.578 | 0.274 | 0.732 |
|  | HAP mean | 0.685 | 0.470 | 0.407 | 0.762 |
|  | SNP mean | 0.669 | 0.494 | 0.388 | 0.720 |
|  | PC max | 0.731 | 0.711 | 0.585 | 0.820 |
|  | HAP max | 0.731 | 0.681 | 0.616 | 0.835 |
|  | SNP max | 0.734 | 0.711 | 0.578 | 0.834 |

**Table S5** Predictive abilities (PAs) of four test populations for seed yield (YLD) under across-population prediction (APP) using CORE378 as the training population

| Model | Marker representation | Test population | | | |
| --- | --- | --- | --- | --- | --- |
|  |  | BP295 | YS38 | BS61 | BMEVSU260 |
| R_RRBLUP | PC | 0.720 | 0.598 | 0.451 | 0.703 |
|  | HAP | 0.737 | 0.446 | 0.500 | 0.704 |
|  | SNP | 0.728 | 0.600 | 0.493 | 0.697 |
| R_GBLUP | PC | 0.664 | 0.664 | 0.494 | 0.789 |
|  | HAP | 0.673 | 0.608 | 0.565 | 0.765 |
|  | SNP | 0.664 | 0.664 | 0.494 | 0.790 |
| BRR | PC | 0.720 | 0.597 | 0.451 | 0.703 |
|  | HAP | 0.737 | 0.446 | 0.500 | 0.704 |
|  | SNP | 0.728 | 0.599 | 0.493 | 0.697 |
| ElasticNet | PC | 0.726 | 0.626 | 0.444 | 0.749 |
|  | HAP | 0.737 | 0.485 | 0.588 | 0.708 |
|  | SNP | 0.732 | 0.490 | 0.555 | 0.701 |
| RFR | PC | 0.740 | 0.554 | 0.435 | 0.612 |
|  | HAP | 0.716 | 0.247 | 0.553 | 0.673 |
|  | SNP | 0.728 | 0.224 | 0.531 | 0.694 |
| LightGBM | PC | 0.717 | 0.595 | 0.500 | 0.652 |
|  | HAP | 0.716 | 0.231 | 0.535 | 0.673 |
|  | SNP | 0.696 | 0.245 | 0.579 | 0.691 |
| XGBoost | PC | 0.700 | 0.202 | 0.438 | 0.686 |
|  | HAP | 0.675 | 0.214 | 0.553 | 0.586 |
|  | SNP | 0.668 | 0.435 | 0.516 | 0.672 |
| MLPGS | PC | 0.735 | 0.320 | 0.267 | 0.645 |
|  | HAP | 0.732 | 0.271 | 0.181 | 0.596 |
|  | SNP | 0.744 | 0.380 | 0.009 | 0.609 |
| DNNGS | PC | 0.707 | 0.623 | 0.572 | 0.715 |
|  | HAP | 0.767 | 0.515 | 0.481 | 0.758 |
|  | SNP | 0.752 | 0.504 | 0.544 | 0.680 |
| GraphConvGS | PC | 0.533 | 0.289 | -0.655 | 0.658 |
|  | HAP | 0.422 | 0.528 | -0.350 | 0.824 |
|  | SNP | 0.473 | 0.531 | -0.583 | 0.842 |
| GraphAttnGS | PC | 0.587 | 0.245 | -0.268 | 0.662 |
|  | HAP | 0.561 | 0.005 | -0.276 | 0.833 |
|  | SNP | 0.596 | -0.271 | 0.233 | 0.772 |
| GraphSAGEGS | PC | 0.685 | 0.338 | 0.243 | 0.698 |
|  | HAP | 0.742 | 0.091 | 0.145 | 0.743 |
|  | SNP | 0.742 | 0.181 | 0.140 | 0.747 |
| GraphFormer | PC | 0.687 | 0.325 | 0.304 | 0.406 |
|  | HAP | 0.735 | 0.118 | 0.047 | 0.735 |
|  | SNP | 0.726 | 0.161 | 0.018 | 0.719 |
| DeepResBLUP | PC | 0.731 | 0.565 | 0.525 | 0.653 |
|  | HAP | 0.713 | 0.446 | 0.375 | 0.713 |
|  | SNP | 0.745 | 0.580 | 0.470 | 0.639 |
| DeepBLUP | PC | 0.665 | 0.552 | 0.436 | 0.696 |
|  | HAP | 0.737 | 0.407 | 0.514 | 0.687 |
|  | SNP | 0.763 | 0.570 | 0.528 | 0.673 |
| EnsembleGS | PC | 0.711 | 0.575 | 0.496 | 0.637 |
|  | HAP | 0.706 | 0.190 | 0.527 | 0.650 |
|  | SNP | 0.675 | 0.187 | 0.570 | 0.684 |
|  | PC mean | 0.689 | 0.479 | 0.321 | 0.667 |
|  | HAP mean | 0.694 | 0.328 | 0.340 | 0.710 |
|  | SNP mean | 0.698 | 0.380 | 0.349 | 0.707 |
|  | PC max | 0.740 | 0.664 | 0.572 | 0.789 |
|  | HAP max | 0.767 | 0.608 | 0.588 | 0.833 |
|  | SNP max | 0.763 | 0.664 | 0.579 | 0.842 |

**Table S6** Predictive abilities (PAs) of two test populations for seed yield (YLD) under across-population prediction (APP) using BP295 as the training population

| Model | Marker representation | YS38 | BS61 |  |
| --- | --- | --- | --- | --- |
| R_RRBLUP | PC | 0.726 | 0.570 |  |
|  | HAP | 0.731 | 0.508 |  |
|  | SNP | 0.728 | 0.571 |  |
| R_GBLUP | PC | 0.439 | 0.503 |  |
|  | HAP | 0.841 | 0.652 |  |
|  | SNP | 0.848 | 0.665 |  |
| BRR | | PC | 0.725 | 0.568 |
|  | | HAP | 0.730 | 0.504 |
|  | | SNP | 0.453 | 0.475 |
| ElasticNet | PC | 0.717 | 0.610 |  |
|  | HAP | 0.674 | 0.666 |  |
|  | SNP | 0.654 | 0.659 |  |
| RFR | PC | 0.424 | 0.125 |  |
|  | HAP | 0.555 | 0.564 |  |
|  | SNP | 0.635 | 0.664 |  |
| LightGBM | PC | 0.595 | 0.076 |  |
|  | HAP | 0.755 | 0.561 |  |
|  | SNP | 0.623 | 0.670 |  |
| XGBoost | PC | 0.513 | 0.290 |  |
|  | HAP | 0.383 | 0.450 |  |
|  | SNP | 0.462 | 0.457 |  |
| MLPGS | PC | 0.745 | 0.643 |  |
|  | HAP | 0.711 | 0.153 |  |
|  | SNP | 0.775 | 0.262 |  |
| DNNGS | PC | 0.740 | 0.588 |  |
|  | HAP | 0.273 | 0.668 |  |
|  | SNP | -0.076 | 0.136 |  |
| GraphConvGS | PC | 0.669 | 0.379 |  |
|  | HAP | 0.688 | -0.373 |  |
|  | SNP | 0.670 | 0.477 |  |
| GraphAttnGS | PC | -0.237 | 0.511 |  |
|  | HAP | -0.560 | 0.546 |  |
|  | SNP | 0.217 | 0.362 |  |
| GraphSAGEGS | PC | 0.720 | 0.589 |  |
|  | HAP | 0.726 | 0.272 |  |
|  | SNP | 0.769 | 0.214 |  |
| GraphFormer | PC | 0.729 | 0.319 |  |
|  | HAP | 0.774 | 0.380 |  |
|  | SNP | 0.701 | 0.242 |  |
| DeepResBLUP | PC | 0.798 | 0.539 |  |
|  | HAP | 0.614 | 0.386 |  |
|  | SNP | 0.633 | 0.474 |  |
| DeepBLUP | PC | 0.719 | 0.574 |  |
|  | HAP | 0.674 | 0.340 |  |
|  | SNP | 0.727 | 0.237 |  |
| EnsembleGS | PC | 0.720 | 0.453 |  |
|  | HAP | 0.790 | 0.500 |  |
|  | SNP | 0.525 | 0.530 |  |
|  | PC mean | 0.609 | 0.459 |  |
|  | HAP mean | 0.585 | 0.424 |  |
|  | SNP mean | 0.584 | 0.443 |  |
|  | PC max | 0.798 | 0.643 |  |
|  | HAP max | 0.841 | 0.668 |  |
|  | SNP max | 0.848 | 0.670 |  |
