# Supplementary material for: Genomic selection for seed yield enhances flax breeding efficiency
Source: Mol Breed. 2026 Jun 2;46(6):61. doi: 10.1007/s11032-026-01684-3 (PMC13230323; doi:10.1007/s11032-026-01684-3)
Supplement: Supplementary file 2 — Supplementary Material 2 (figures) [file 11032_2026_1684_MOESM2_ESM.docx]

**Supplementary Figures**

**
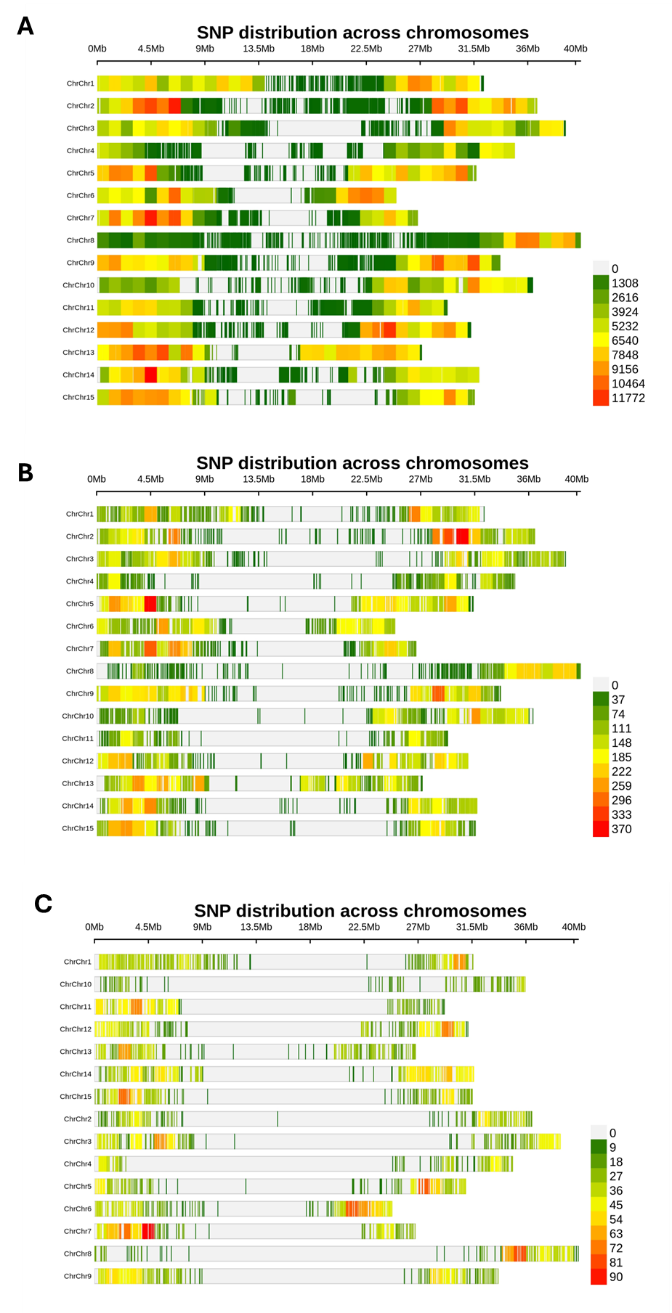
**

**Fig. S1** SNP density along chromosomes (A) in the core collection of 378 accessions (CORE378) comprising ~1.7M SNPs, (B) 33,596 SNPs shared between CORE378 and the 260 test lines from the combined biparental population BMEVSU260, and (C) 5,604 SNPs shared by CORE378, CORE293, YS38, BS61 and BP295. Plots were generated with a 100-Kb window using the CMplot R package (Yin et al. 2021)


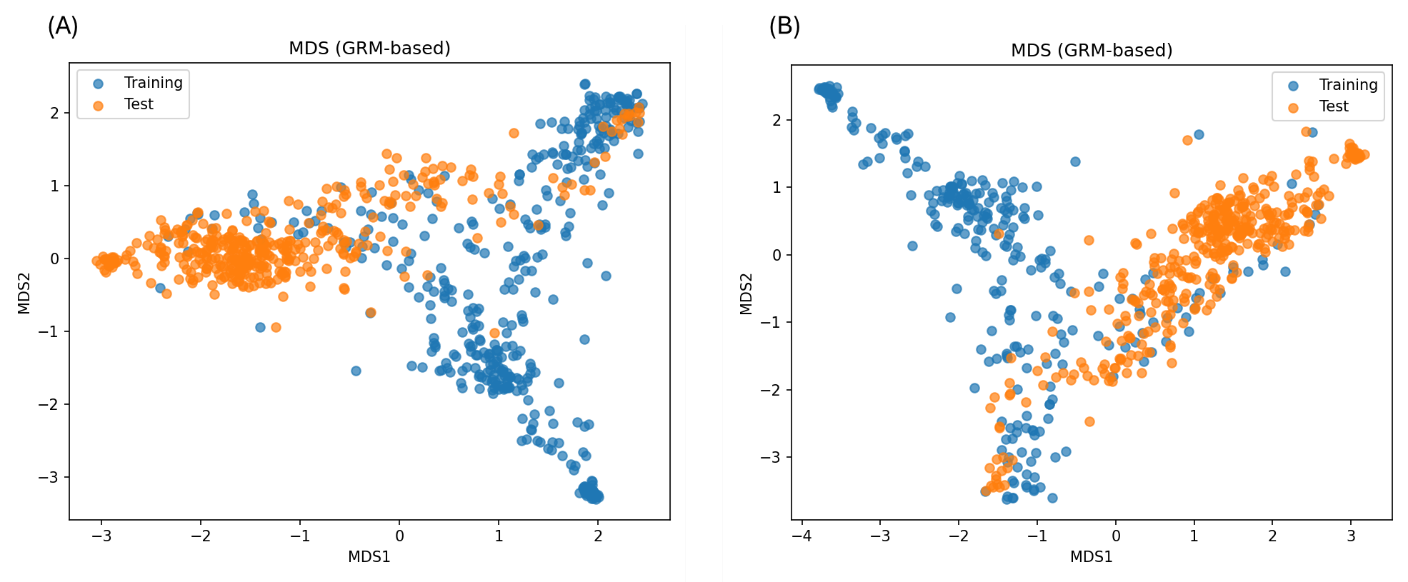


**Fig. S2** Genetic relationships between training and test populations based on GRM-derived multidimensional scaling (MDS). (A) CORE378 (training; blue) versus BP295 (test; orange). (B) CORE293 (training; blue) versus BP295 (test; orange). Dots represent individual genotypes projected onto the first two MDS axes derived from the genomic relationship matrix (GRM). In panel (A), CORE378 shows broader genetic coverage and greater overlap with BP295, whereas in panel (B), CORE293 exhibits more limited overlap with BP295
